# A more equal deal? Employer-employee flexibility, gender and parents’ work-family tensions in Sweden

**DOI:** 10.3233/WOR-210668

**Published:** 2022-11-11

**Authors:** Anne Grönlund, Ida Öun

**Affiliations:** a Department of Social work, Umeå University, Umeå, Sweden; b Department of Sociology, Umeå University, Umeå, Sweden

**Keywords:** Flexibility, schedule control, gender, organizational demands, work-family reconciliation

## Abstract

**BACKGROUND:**

The potential of flexible scheduling to alleviate work-family tensions and replace female part-time work has not been thoroughly explored. Specifically, research has not acknowledged that employees’ schedule control may be conditioned by organizational demands for availability and commitment.

**OBJECTIVE:**

We examine the links between flexibility and gendered patterns of work-family reconciliation by considering how work arrangements balance employer demands and employee control and how they relate to work-family tensions.

**METHODS:**

Using mixed-methods, we combine a survey of Swedish parents (*n* = 2320) with interviews of survey respondents (*n* = 40). First, we identify clusters of flexible work arrangements and explore differences between mothers and fathers. Second, we analyze the relationship between flexible work arrangements and work-family tensions. Finally, the qualitative data are used to explore how flexibility/lack of flexibility enter into parents’ work-family tensions and negotiations.

**RESULTS:**

Three types of flexible work arrangements are found. Boundaryless jobs, which combine high levels of control with high requirements for organizational flexibility, are more common among fathers and highly educated. Confined jobs have low levels of both employee- and employer-oriented flexibility, but high demands, and are common among mothers and in female-dominated workplaces. Despite higher levels of control, boundaryless jobs are not associated with less work-family conflict. In malleable jobs, control is relatively high and demands low and work-family tensions are less noticeable.

**CONCLUSIONS:**

Employer- and employee-oriented flexibility go hand in hand, but work arrangements differ radically between groups. High flexibility does not alleviate work-family tensions, and part-time work remains an important work-family strategy for mothers.

## 1 Introduction

Schedule flexibility has been widely promoted as a ‘family-friendly’ work arrangement that will alleviate work-family tensions and allow parents to share paid work and care more equally. Meanwhile, the relations between flexibility and work-family conflict seem unclear, while gender patterns remain salient. In particular, it is not obvious that schedule flexibility could replace female part-time work as a strategy for work-family reconciliation. To better understand the links between flexibility, gender and work-family tensions, researchers should recognize that flexibility is also an organizational feature and that employees’ schedule control may be conditioned by high demands for availability and commitment. Below, we explore these propositions, using a study of Swedish parents.

In Europe, flexible scheduling defined as schedule control, that is, employees’ possibilities to influence the timing of their work [cf. 1], has become more prevalent and is particularly widespread in Sweden and other Nordic countries [2]. Clearly, however, trends towards flexible working are not driven only by demands from overburdened parents, but more fundamentally by new ways of organizing work. The concept of labour market flexibilization can be defined as a de-regulation and de-standardisation of work, resulting from mega-trends such as globalisation, digitalization, service sector growth and demographic developments [3, cf. 4]. Here, just-in-time productions systems based on worldwide supply chains and an increase in operating hours of service establishments has prompted employers to adapt work hours to deal with fluctuations in demand. These flexibilization trends are broadly discussed as a ’win-win’ concept for both parties, yet it is not obvious that employers’ needs to engage labour more flexibly will facilitate employees’ work-family reconciliation [3]. To explore these issues, a gender perspective is crucial. The de-standardisation of working time is often regarded as an opportunity to increase female labour force participation, but in practice this has been achieved largely through part-time employment [3]. An interesting question, then, is whether more schedule control could provide an alternative to part-time and pave the way for a more gender-equal sharing of paid and unpaid work.

Since time is contested terrain in employer-employee relations, the balancing of interests is crucial for understanding working time flexibilization. In studies developing typologies of flexible working time arrangements a main distinction is made between employer-oriented flexibility serving the needs of the organization and employee-oriented flexibility catering to the needs of workers [for an overview, see 5]. Some studies discuss the employee- versus employer-orientation at opposite ends of a linear continuum, whereas others regard them as separate dimensions [5], implying that employer- and employee-oriented forms of flexibility can be combined. Presumably, however, combinations can vary between countries and groups of workers. Connecting working time flexibilization with national labour market institutions, Berg, Bosch and Charest [6] identify three ideal types. In the mandated configuration, exemplified by France in the study, the strong role of the state has ensured that working time practices remain standardised across the economy, with little flexibility for firms and employees. In the unilateral configuration, typified by the US, flexibilization has been driven by employers and resulted in fragmented and volatile working time patterns, with individual options only for groups with high bargaining power. Finally, Sweden is a prime example of the negotiated configuration, where collective agreements between social partners have supported the creation of a new, more flexible working time, with considerable room for firm-level negotiations. These agreements are described as “compromises that balance employee and employer interest” [6:832]; however, the content of such compromises will vary across industries, workplaces and jobs, with different implications for different groups.

Several scholars have argued that schedule control may be introduced as a company strategy to increase employees’ motivation, loyalty and performance, rather than to cater to their family concerns [1, 7–9]. The argument is supported by studies showing positive correlations between employees’ schedule control and their employers’ demands for working time flexibility in terms of unpaid overtime, work hours varying with fluctuations in work load, frequent business travels and requirements for constant availability [7, 10, 11]. Notably, women report less schedule control than men [11, 12] but also lower demands for employer-oriented flexibility [11, 13, 14]. Further, studies indicate that both types of flexibility are more frequent in high-skilled jobs, but also the gender composition of occupations and workplaces appears to matter [1, 11]. In sum, employee- and employer-oriented flexibility may be closely intertwined, yet we do not know how different arrangements relate to work-family tensions and strategies in dual-earner families.

Individuals’ possibilities to balance demands from work and family can be assessed with the construct of work-family conflict, which has a dominating standing in work-family research. Work-family conflict, or more precisely work-to-family conflict,^1^ captures subjective experiences of work spilling over on family life, creating a tangible stress with negative consequences, such as depression and anxiety, burnout, absenteeism, less job and life satisfaction and worse parent-child relationships [15]. Typically, the number of work hours – a main indicator of demands from the work sphere – is a strong predictor of conflict, while the importance of flexibility – measured as employee influence over the timing of work – is less clear. In meta-analyses [16–19], flexible schedules tend to be negatively correlated with work-family conflict; however, as Allen et al. [17] point out, effects are small and in some studies, flexibility is associated with higher work-family conflict [20].

In our view, these incongruent findings and limited ‘net’ effects call for a more comprehensive view of flexibility. First, it should be recognized that flexibility is also an organizational demand. To date, however, employer requirements for schedule flexibility have been little explored in research on work-family conflict. Second, the strain dimension has not been sufficiently considered.^2^ Arguably, schedule control may not prevent strain-based negative spillover from intensive work in downsized organizations, and empirical studies suggest that schedule control is connected to longer work hours and higher work intensity, at least for men [7, 21]. Meanwhile, research on work-family conflict has retained a strong focus on time and when measures of strain are included they tend to capture employer demands [for overviews, see 16, 18] but not employee control. This conception of strain deviates from that found in research on work and stress, where the job demand-control model [22, 23] argues that the consequences of job demands are crucially affected by employees’ level of control, defined as their possibilities to influence the pace of work and decide when and how to perform different tasks [for overviews of empirical findings, see 24]. To get a fuller picture, our analysis comprises employer demands and employee control in both time and strain dimensions. (Please note that we use the terms psychological demands and task control for the job demands/job control measures. This is to distinguish them from the time dimension, which is not considered in this model.)

To understand gender patterns in reported work-family conflict, we must also consider the strategies employed to keep such conflicts down. Part-time work has been as a main tool for women and mothers^3^ to combine paid work and care, yet this strategy is far from unproblematic. Generally, part-time work entails worse labour market outcomes and larger burdens in terms of housework and childcare [25, 26], and at a time when women invest heavily in higher education, such a strategy may be less viable and desirable. Potentially, more flexible scheduling could provide a new alternative that would allow women and men to engage in full-time work while taking turns doing housework and childcare [27]. To study the importance of flexibility from a gender perspective, we go beyond a strictly variable-oriented approach to also consider contexts. Clearly, both the work-family tensions and the strategies available to offset them will differ for women (and men) in countries with different norms and institutions and, as explained below, Sweden provides a good case for our study. However, also jobs will be regarded as contexts characterized by different working conditions, as we identify clusters of jobs differing in employer- and employee-oriented flexibility. Apart from the theoretical deliberations described above, this is a methodologically relevant approach which has been little used in the study of flexibility and work-family tensions. As argued by Härenstam [28], contextual and comprehensive analyses, such as cluster analyses, “are particularly suitable for comparisons of women’s and men’s risk factors for health in working life as well as for guidance in preventive action” as they “help identify the gender-structured situations for women and men and facilitate the discovery of differences among all women and all men.” Finally, the family provides an important context where gendered roles and responsibilities are negotiated, within the framework of societal norms and institutions [29] as well as demands and opportunities stemming from mothers’ and fathers’ jobs.

The Swedish context provides a strong test for assessing the gender-equalizing potential of flexibility. Sweden boasts high rates of female and maternal labour force participation [30] and these achievements are commonly attributed to the extensive family policies. The policy package comprises generous parental leaves and a right to low-cost public day-care, but also specific tools intended to alleviate the daily frictions between work and family, specifically statutory rights for parents to reduce work hours and to take paid leave to care for sick children (hereafter: CSC-leave).^4^ Despite these provisions, however, work-family conflict is comparatively high from a European perspective [31, but see 32], particularly for mothers. Even in Sweden, women take more responsibility for housework and care than men and fare worse in terms of wages and careers, and parenthood tends to widen these gaps [14, 33, 34]. These tensions suggest that traditional family policies may not be sufficient in the transition from a 1.5-earner society to a gender equal dual-earner society. Arguably, flexibility could provide a new tool and in Sweden, schedule control is widely available to employees [2, cf. 6]^5^ As discussed, however, schedule control may come with organizational demands that could hamper its ‘family-friendliness.’ Also, high levels of flexibility could be a source of conflict because individuals are required to define and structure their work and draw the line between work and non-work [4]. In this situation, statutory rights to work hour reductions and CSC-leave could also be difficult to use [10, 35].

Below, we examine these issues using a recent survey of Swedish parents supplemented by interviews with survey respondents. We identify different structures of flexible work arrangements and how they differ for mothers and fathers. Next, we analyze the relationship between flexible work arrangements and work-family tensions, both overall levels of work-family conflict and conflicts arising when children are sick. Here, we can assess the usefulness of flexible work arrangement in relation to other family policy tools, notably CSC-leave, but also part-time work. Finally, the qualitative data are used to explore how flexibility - or lack of flexibility - enter into mothers and fathers work-family tensions and negotiations.

## 2 Aim and research questions

The aim of the study is to examine the links between flexible scheduling and gendered patterns of work-family reconciliation by considering how work arrangements balance employer demands and employee control and how they relate to parents’ work-family tensions.

First (RQ 1), we ask how flexible work arrangements are structured in terms of employer demands and employee control, and how patterns of flexibility vary between mothers and fathers.

Next (RQ 2), we analyze how patterns of flexibility are related to parents’ daily work-family tensions. We measure both work-to-family conflict and specific conflicts arising when children are sick, namely problems in taking formal leave to care for sick children [CSC-problems] and parents’ propensity to work while caring for a sick child [WSC-practices]. Also, we ask whether full-time work implies less work-family tensions for employees with flexible work arrangements and if flexibility provides an equal alternative to part-time work.

Finally (RQ 3), we use the qualitative data to explore how flexibility is perceived and handled in parents’ work-family negotiations and tensions.

## 3 Data and methods

The study presented here is based on a mixed methods approach, specifically a sequential explanatory QUANT-qual study [36]. This means that the data collection started with the quantitative data, which later was complemented by qualitative data. In the analysis, priority was given to the quantitative data while the qualitative data are used to further interpret the findings. A major strength of such a design is the possibility to “simultaneously confirm a quantitatively derived hypothesis and explore in greater depth the processes by which the relationship occurred” [16, 37]. Below, we will describe the two datasets and how each of them was analysed.

### 3.1 Quantitative data and analysis

The quantitative data comes from a postal survey completed in 2016. Sampling, distribution, and coding were administered by Statistics Sweden. The sample was a simple random sample drawn from the Swedish Register of the total population and comprised 5 000 parents whose youngest child was 3–6 years old. The sampling strategy was motivated by our focus on flexible work arrangements and work-family tensions among parents of young children. For this reason, we included parents whose youngest child had not yet started school but excluded families in which one parent could still be on parental leave.^6^ Here, we use a subsample comprising employed mothers and fathers (n ≈ 2 320).

To answer RQ 1, our first step was to identify patterns of flexible work arrangements using Latent Class Analysis (LCA). LCA is a method that enables us to retrieve nonlinear relationships between categorical variables in order to identify qualitatively different configurations of flexible/non-flexible work arrangements. In LCA, the notion of local independence is central: the method examines whether relationships within a set of observed indicators are explained by latent clusters [38, 39]. LCA distinguishes dominant patterns of flexible and non-flexible work arrangements in the data, and all respondents sharing similar patterns are allocated to a specific cluster. Thus, the objective of this data-reduction method is to identify groups of individuals who share similar characteristics. For example, if two dominant patterns of work arrangements exist among the respondents, a two-cluster model will fit the data. If the sample can be divided into three main configurations of work arrangements, a three-cluster model will be selected, and so on. By applying different model fit statistics, the number of dominant clusters can be determined.

The idea was to examine how the respondents’ jobs were structured in terms of employer demands and employee control, both in the time and strain dimensions [cf. 28]. Here, four variables were used. *Schedule control* measures the degree to which the respondent can influence the timing (start and ending time) of his/her work. The survey offered four options (a) no influence, fixed schedule, b) can move start/ending time up to one hour, c) can move it a couple of hours, or d) can move it a day or more. Meanwhile, *flexibility demands* captures the requirements for schedule variability from the employer. This is an index comprising three items “The number of hours I work varies considerably as the work load varies”, “My work hours are too unpredictable”, “I am expected to be available by phone or email during non-work hours” (range 3–12, mean 6.4, std. 2.6, median 6.0, alpha 0.76). This measure captures central aspects of employer oriented flexibility identified in previous studies [5, 11, 12]. For the strain dimension we rely on the established measures from the job demand-control model [23], but distinguish them from our time-related items by using the terms psychological demands for job demands and task control for job control. *Psychological demands* are captured with an index of two indicators, “My work is psychologically demanding” and “Due to a high workload I often work under great time pressure” (range 2–10, mean 6.4, std. 2.0, median 6.0, alpha 0.69). *Task control* combines four items capturing the respondent’s degree of control over a) which work tasks to perform, b) the way to perform work tasks, c) work pace, and d) important decisions concerning the work place (range 4–20, mean 13.9, std. 3.5, median 14.0, alpha 0.83). In the LCA, all variables were dichotomized into low and high levels of demands and control based on cut-points from sample medians. High schedule control was defined as being able to more working time by a day or more.

LCA also calculates the probability of each individual to belong to each cluster and in the next step, we used cluster membership probabilities to examine how the different types of flexible (or non-flexible) work arrangements were distributed among the respondents. The analysis was based on ordinary least square regressions, OLS, in which the LCA clusters were used as dependent variables. Our main independent variable in this analysis is the respondents’ *gender.* Based on findings from previous studies, in a second step, we also control for *education* (university/non-university), and, finally, we introduce *workplace gender composition* (more men/mixed/more women).

To address RQ 2, we performed linear regressions with three dependent variables. *Work-to-family conflict (WFC)* is an additive index of four questions asking how often the respondent (a) keeps worrying about work problems when he/she is not working (b) feels too tired after work to enjoy the things he/she would like to do at home (c) finds that his/her job prevents him/her from giving the time he/she wants to his/her partner or family (d) feels that his/her partner/family becomes fed up with the pressure from his/her job (five response categories, index range 0–100, higher values means more conflict, mean 48.9, std 19.1). This is an established measure of work-to-family conflict (WIF) applied in many contexts [16, 18, 31, 40]. To measure specific conflicts arising when children are sick we used two dichotomous variables: *CSC-problems* measure whether or not the respondent feels that using the formal leave to care for children (see above) more than occasionally causes problems at his/her workplace, while *WSC-practices* indicate whether or not the respondent works from home while caring for sick children (response categories: fully agree, agree to some extent, disagree to some extent, fully disagree – dichotomized into agree and disagree; 41.0 % of the respondents experience CSC-problems, 43.4% experience WSC-practices). To analyze the probability for CSC-problems and WSC-practices, linear probability models were estimated. For WFC, OLS regressions were used. In the first model, the independent variable was cluster-gender groups. This variable was based on the cluster groups retrieved in the LCA; however, for each cluster we also distinguished between mothers and fathers. In the second model, we added *work hours,* categorized into part-time (<35 hours per week), full-time (35–40 hours), and long work hours (>40 hours). By controlling for work hours in the second model, we examined how experiences of WFC, CSC-problems and WSC-practices relate to working time. More specifically, we wanted to see whether full-time work implies less work-family tensions for employees with flexible work arrangements. By adding work hours, we also examined whether and how the relationships between gender-cluster groups and work-family tensions are influenced by work hours. Finally, we introduced interaction variables (work hours*clusters) to capture how the impact of working time varies between clusters for mothers and fathers and determine whether flexibility provides an equal alternative to part-time work. Results for the interaction analysis are presented as predicted values in a separate table.

### 3.2 Qualitative data and analysis

Data for the qualitative analysis come from 40 interviews with respondents that took part in the survey described above. In the survey, 900 individuals agreed to be contacted for interviews; thus, we were able to strategically select interview respondents on the basis of gender and educational level. The final sample included 20 mothers and 20 fathers. Twenty-six of the respondents had a university degree, the others did not. Also, there was variation in family type, geographical location, number of children and occupation. Selected demographics for interview respondents can be found in Table 1.

**Table 1:** Demographics of the interview respondents

	Education		Marital status^1^		Number of children		
	University	No university	Married	Single	One	Two	Three <
Mothers	12	8	16	4	5	10	5
Fathers	14	6	17	3	5	8	7
All	26	14	33	7	10	18	12

^1^Single means that the respondent is separated from the other parent of the youngest child. Married includes cohabiting parents.

The interviews were conducted via the internet during winter and spring 2018. They lasted on average 60 minutes and were audio recorded and transcribed. The interview guide included questions about the use and usefulness of family policy rights (i.e. day care, part-time work, parental leave and CSC-leave) as well as parents’ perceptions of conflict and strategies in work-family arrangements.

The qualitative analysis aimed at gaining a deeper understanding of the quantitative results by exploring how flexibility was perceived and handled in parents’ work-family negotiations (RQ 3). The analysis was based on thematic analysis [41]. With this method, the researcher performs an initial coding of the data, then proceeds to identify themes – that is, patterns of responses or meaning – in the data. Next, the researcher creates thematic maps, connecting the themes to each other and the research question, and finally, all themes must be scrutinized to make sure they are clearly defined and firmly based on the data.

## 4 Results

Below, we first present the results from the quantitative analysis (RQ 1-2), and then the qualitative findings (RQ 3).

### 4.1 Quantitative findings

The first research question (RQ 1) asks what types of flexible work arrangements can be discerned in the data and how they are distributed across groups. Latent class analysis was used to identify clusters of jobs with similar properties in terms of employee control and organizational demands in both time and strain dimensions. The best fit for the data was the three-cluster model displayed in Table 2 (for model fit statistics, see Supplementary Table 1). As shown, two clusters are equally large, each comprising 37-38 percent of the respondents. The third cluster is smaller, representing 25 percent of the respondents.

**Table 2:** Characteristics of three types of flexible work arrangements. Latent class analysis

	Cluster 1	Cluster 2	Cluster 3
	Confined jobs	Boundaryless jobs	Malleable jobs
Schedule control
Low	0.77	0.19	0.49
High	0.23	0.81	0.51
Task control
Low	0.84	0.28	0.57
High	0.16	0.72	0.43
Flexibility demands
Low	0.78	0.25	0.99
High	0.22	0.75	0.01
Psychological demands
Low	0.35	0.42	0.91
High	0.65	0.58	0.09
Cluster size % of sample	37.8	37.2	24.9

Work arrangements in the first cluster – the *confined jobs* – are characterized by low levels of both schedule and task control. While organizational requirements are high in terms of psychological demands, flexibility demands from the employer are low. Lacking both schedule and task control, employees are confined and left with limited room for maneuver. Further, the work tasks are confined in time and space, as indicated by the low levels of flexibility demands. The *boundaryless jobs* in cluster two provide a stark contrast. Here, employees have high levels of both schedule and task control. At the same time, organizational requirements are high both in terms of psychological demands and flexibility demands. These characteristics imply a boundaryless situation where employees are both allowed and required to define and delimit their work and, consequently, to negotiate the boundaries between work and non-work roles. The third cluster consists of *malleable jobs.* Respondents in this cluster report low psychological demands as well as low flexibility demands from the employer. Regarding the levels of schedule and task control for the employee, the cluster seems divided. However, further analyses show that no one in this cluster has low levels of both schedule and task control, and that some have high levels of both. These jobs can be regarded as regulated but malleable since the combination of low demands and high schedule and/or task control provide employees with some space for adjusting both work pace and schedules.

To examine how flexible work arrangements differ across groups, we conducted OLS regressions using the clusters as dependent variables. The results, displayed in Table 3, show that boundaryless jobs are more common among fathers, high-educated parents and in gender-mixed and, particularly, male-dominated workplaces. Confined jobs are more common among mothers, parents with non-tertiary education and in female-dominated workplaces. The malleable jobs, too, are held mainly by parents with non-tertiary education and those working in female-dominated workplaces; however, the differences between educational groups and workplaces are smaller than for the other clusters and mothers and fathers are equally likely to have a malleable job.^7^ We should note that work hour patterns vary both between clusters and between mothers and fathers. As shown in Supplementary Table 2, 52 percent of the fathers and 37 percent of the mothers in boundaryless jobs work long hours. In the other clusters, long hours are considerably less common, though they are more common among fathers than among mothers. Meanwhile, only 14 percent of mothers in boundaryless jobs work part-time, compared to 35 and 26 percent in confined and malleable jobs, respectively. Fathers working part-time is uncommon in all clusters.

**Table 3:** Cluster probabilities (%) for three types of flexible work arrangements by gender, education and gender composition of workplace (*n* = 2014)

	Cluster 1 Confined jobs		Cluster 2 Boundaryless jobs		Cluster 3 Malleable jobs	
	*b*	*s.e.*	*b*	*s.e.*	*b*	*s.e.*
Intercept	**41.15**	0.80	**31.96**	0.90	**26.88**	0.74
Gender
Men	**–3.87**	0.78	**3.33**	0.88	0.55	0.73
Women	**3.87**	0.78	**–3.33**	0.88	–0.55	0.73
Education
No degree	**4.82**	0.80	**–6.93**	0.91	**2.11**	0.75
University degree	**–4.82**	0.80	**6.93**	0.91	**–2.11**	0.75
Workplace gender composition
More men	**–4.70**	1.06	**7.15**	1.20	**–2.44**	0.99
Mixed	**–2.62**	1.02	**3.22**	1.15	–0.59	0.95
More women	**7.33**	1.02	**–10.36**	1.15	**3.04**	0.95
R^2^ (%)	6.9	8.6	0.9

Multiple OLS regression. Cell entries are unstandardized regression coefficients*100 and standard errors*100. Bold coefficients = significantly different from zero (*p* < 0.05).

To answer RQ 2, we used linear regressions to study how cluster and gender groups vary in terms of a) parents’ perceptions of generating workplace problems by taking leave to care for sick children (CSC-problems), b) their propensity to work from home while caring for a sick child (WSC-practices) and c) their level of work-family conflict (WFC). As shown in Table 4, CSC-problems (model 1a) are reported both in confined jobs and in boundaryless jobs, but much less often in malleable jobs. In confined jobs, mothers are more likely to perceive CSC-problems, while in boundaryless jobs such problems are more often reported by fathers. WSC-practices (model 2a) are by far most widespread in boundaryless jobs and, within this cluster, they are more common among mothers than fathers. In the malleable jobs and, particularly, in confined jobs, WSC-practices are less common and gender differences are smaller. Regarding WFC (model 3a), mothers in boundaryless jobs stand out with a level of work-family conflict that is considerably higher than for men in the same cluster but also higher than for mothers in the other clusters. Gender differences are found in all clusters, however in malleable jobs, both mothers and fathers perceive much less conflict is than in the other clusters.

**Table 4:** CSC-problems, WSC-practices and WFC by cluster-gender groups and work hour categories

	CSC-problems				WSC-practices				WFC			
	Model 1		Model 2		Model 3		Model 4		Model 5		Model 6	
	*b*	*s.e.*	*b*	*s.e.*	*b*	*s.e.*	*b*	*s.e.*	*b*	*s.e.*	*b*	*s.e.*
Intercept	**38.78**	1.08	**40.76**	1.24	**44.98**	0.96	**44.68**	1.07	**47.37**	0.40	**47.21**	0.45
Cluster-gender groups
Confined W	**11.62**	1.91	**13.72**	1.98	**–21.81**	1.70	**–16.30**	1.73	**5.44**	0.71	**7.72**	0.72
Confined M	**6.53**	2.45	**7.48**	2.45	**–23.86**	2.18	**–24.39**	2.12	–0.56	0.91	–0.66	0.89
Boundaryless W	**6.00**	2.43	3.31	2.44	**36.67**	2.16	**33.44**	2.12	**10.08**	0.91	**8.71**	0.89
Boundaryless M	**9.52**	2.22	4.19	2.33	**30.69**	1.97	**22.78**	2.02	**4.63**	0.83	1.18	0.85
Malleable W	**–15.51**	2.60	**–12.48**	2.62	**–10.92**	2.32	**–6.19**	2.27	**–8.03**	0.97	**–5.97**	0.95
Malleable M	**–18.17**	2.82	**–16.22**	2.83	**–10.77**	2.51	**–9.34**	2.46	**–11.56**	1.05	**–10.96**	1.03
Work hours
<35 h/w (part-time)			**–5.62**	1.95			**–16.94**	1.69			**–6.92**	0.71
35–40 h/w (full-time)			**–6.88**	1.42			**–3.14**	1.24			**–1.28**	0.52
>40 h/w (long work hours)			**12.50**	1.81			**20.08**	1.57			**8.21**	0.66
R^2^ (%)	5.0		7.4		25.3		30.8		12.8		18.6
N	2188		2161		2194		2167		2182		2156

Multiple OLS regression. Cell entries are unstandardized regression coefficients*100 and standard errors*100. Bold coefficients = significantly different from zero (*p* < 0.05).

Regarding work hours, we note that CSC-problems (model 1b) are particularly common for parents with long work hours, as are WSC-practices (model 2b) and WFC (model 3b). However, for WSC-practices and WFC there is also a clear difference between full-time and part-time work. When work hours accounted for, the differences between clusters decrease, but patterns of change vary both between clusters and between mothers and fathers, suggesting a complex interplay between flexibility, work hours and gender.

To examine this further, we conducted gender-separate regressions, including interaction variables that capture how the importance of work hours varies with cluster membership. The results are presented as predicted effects in Table 5. These allow us to assess if full-time work is less problematic in flexible jobs than in other jobs but also compare full-time and part-time work within each cluster. The table shows that, for mothers working full-time, CSC-problems are particularly common in confined jobs, while WSC-practices and WFC are most prevalent in boundaryless jobs. For fathers working full-time, WSC-practices are also most common in boundaryless; however, regarding WFC and CSC-problems, these jobs do not differ from the confined jobs. Compared to full-time workers, mothers working part-time work report less WFC and WSC-practices, but are not less likely to experience CSC-problems. The difference between part-time and full-time work does not vary between clusters. However, working long hours (rather than full-time) makes a larger difference in confined than in boundaryless jobs for mothers’ WFC and for mothers’ and fathers’ WSC-practices. For fathers’, working part-time compared to working full time does not make a difference on any indicator in any cluster.

**Table 5:** Predicted values based on regression with interaction terms work hour categories*clusters. Reference groups: confined jobs, full-time work

CSC -problems	Mothers			Fathers		
	Confined jobs	Boundaryless jobs	Malleable jobs	Confined jobs	Boundaryless jobs	Malleable jobs
Part-time work	47.73	34.88	23.94	59.09	38.46	16.67
Full-time work	48.14	34.59^#^	20.56^#^	42.17	39.44	17.44^#^
Long work hours	67.95^*^	61.34^*^	43.48^*^	52.31	56.34	39.39
WSC-practices
Part-time work	9.55^*^	56.82^*^	22.54^*^	9.09	69.23	27.78
Full-time work	20.87	77.99^#^	35.56^#^	15.15	71.43^#^	32.56^#^
Long work hours	71.79^*^	95.80^†^	54.17^†^	45.45^*^	80.37^†^	48.48^*^
WFC
Part-time work	47.94^*^	46.02^*^	35.39^*^	43.45	41.83	28.47
Full-time work	52.89	56.45^#^	39.46^#^	44.92	47.86	34.90^#^
Long work hours	66.48^*^	62.82^†^	50.00^*^	54.73^*^	55.92^*^	45.27^*^

Statistical significance indications (*p* < 0.05): ^#^cluster difference among full-time workers (ref: confined). ^*^difference between work hour categories within clusters (ref: full-time). ^†^cluster difference in effect of longer/shorter work hours than full-time (interaction term, ref confined). Note: The predicted values are derived from gender-separate regression models with interactions between work hour categories and clusters. In the calculations, all direct effects as well as interaction effects are accounted for.

### 4.2 Qualitative findings

The aim of the qualitative analysis was to deepen our understanding of the quantitative findings by exploring how flexible work arrangements are perceived and handled in parents’ work-family negotiations and tensions (RQ 3).

By structuring the data based on the flexibility of the respondents’ job, we identify three groups corresponding to the clusters described in the section above (boundaryless, confined and malleable jobs). Table 6 summarizes the central themes in each group: the general character of their work-family adaptions, the sharing of paid and unpaid work in the family, the usage of family policy tools (part-time work, CSC-leave) and finally, the specific tensions emerging in the narratives. Obviously, these categories should be regarded as ideal types rather than clear-cut characterizations of individual’s life stories. Instead, the typology could provide a theoretical tool for exploring the mechanisms linking flexibility, gender and work-family reconciliation, as will be further discussed below.

**Table 6:** Typology of flexibility and work-family reconciliation

Job type	Work-family adaptions	Sharing of paid and unpaid work	Policy usage	Tensions
Boundaryless, 12 fathers, 5 mothers	Constant boundary management	* Equal or unequal: Tag-team arrangements where partners share work equally by taking turns in the home while both working full-time. Several fathers have a partner who work part-time and take main responsibility for unpaid work	* Part-time not possible; work hours often extend beyond normal fulltime	* Schedule control as a reward; new form of ideal worker norm?
		* Single parents with shared custody can concentrate work to childless weeks	* WSC-practices instead of using CSC-leave	* Effors to limit family-unfriendly demands e g travelling
				* Partner central; equal sharing crucial for mothers. Grandparents can make a difference
Confined, 8 fathers, 8 mothers	Coping by role specialisation	* Unequal sharing: mothers take main responsibility for unpaid work	* Part-time work main solution, but not always possible. Most fathers never considered part-time.	* Traditional gender roles despite ideals of equality
		*Either role specialisation with full-time/ part-time work or both parents on full-time with second shift for mothers	* CSC-leave possible but not unproblematic.	* Part-time work a way (for mothers) to cope with job strain and care needs. However, economic restraints and job insecurity leave some with fewer options. Grandparents can make a difference
		*Fathers’ employer flexibility require mothers to adapt
Malleable, 0 fathers, 7 mothers	Strategic adaption	* Schedule control used as an alternative to part-time	* Part-time possible but not necessary to deal with care needs	* Partner less crucial but part of solution
		* Relatively equal sharing of unpaid work	* CSC-leave possible but not unproblematic	* Care needs and work stress less pressing than in other groups

For respondents in boundaryless job, the negotiation of work and family demands is characterized by *constant boundary management*. Respondents in this group – clearly dominated by fathers – have jobs entailing high levels of schedule control coupled with high demands for employer flexibility, but these dimensions are not described as incompatible. Instead, employer demands for flexibility are seen as an inherent part of the job while schedule control is perceived as a bonus and the arrangement is generally described as a win-win solution. Oscar argues that the freedom he enjoys is reasonable as long as he ‘delivers’ and is constantly available for customers and colleagues: “I see no problem in answering the phone in the evenings. People know that you are available and that is the price you have to pay for flexibility.” The flexibility bonus is a recognition for being productive and professional and several respondents emphasize that this arrangement enables them to be highly involved in both work and care. In practice, this implies a constant negotiation of work-family boundaries, and in several cases, also gender roles are re-negotiated. For Fredrik and his wife, flexible schedules have enabled them to both work full-time and share care responsibilities by taking shifts in the home in a tag-team fashion. “We have shared everything equally since the day our son was born – parental leaves, sick days, school meetings and the leaving and picking up from daycare. When parents use part-time, the result is often a more gendered division of time, because it is the women who reduce hours and take the responsibility for childcare,” says Fredrik. Such equality contracts are reported by all mothers and some fathers, including single fathers who use their flexibility to focus on work and care on alternate weeks. Others have a partner who accommodates the demands of their jobs by taking the main responsibility for unpaid work. However, such traditional arrangements are not unproblematic as they clash with ideals of equal sharing. Several fathers have made efforts to restrict employer-oriented flexibility and emphasize the importance of having an ‘understanding’ employer with a modern view of fatherhood. Says Anders: “I used to do a lot of overnight business trips but my employer has been accommodating and allowed me to cut down on travelling so I can help out with the children at home.” Nevertheless, respondents in this type of arrangement tend to work long hours, at least periodically. All agree that part-time work is not possible in their jobs and the unregulated situation makes work difficult to delimit: “If you sit eight to five at an [office] there is some social control. I have to exercise that control myself and make sure I whip myself: Can you do more today? Can you deliver more?” These negotiations are particularly delicate when children are sick, as respondents tend to bring work home rather than take CSC-leave, and when available, grandparents are called in.

For respondents with ‘confined’ jobs, the main logic of work-family reconciliation can be described as coping by role specialization. These respondents’ jobs are characterized by a lack of schedule flexibility and here, female part-time work emerges as the main strategy for keeping work-family conflict down. In these cases, the mother take on the main responsibility for housework and childcare, and such role specialization is motivated both by their partners’ higher income and boundaryless work. Maria has settled for seasonal jobs that fit with her husband’s long and unpredictable work hours, though she would have preferred full-time work and she feels uncomfortable as she has to turn down job offers that do not fit his schedule: “Of course, [the employer] wants you to be flexible and help out. But I have to put the family first.” Some mothers express frustration at the uneven division of work. “I think he should work part-time [too] so that we can share [housework],” says Johanna. Others emphasize that part-time work was also a way to cope with both stressful demands in their own job and the care needs of children, particularly when illnesses and neuropsychiatric diagnoses are involved. However, in practice, policy rights are not always uncomplicated to use: “You have the right [to part-time] but it’s a right within quotes, as rents and mortgages don’t pay themselves,” says Josephine, a single mother who also worried that too much CSC-leave could jeopardize her job.

The respondents in the final category – all of which are mothers – have jobs that can be categorized as malleable as they entail some amount of schedule control, but no demands for employer flexibility. Regarding work-family reconciliation, the main theme in this group is that of strategic adaption. Respondents in these jobs have the opportunity to work part-time but see no reason to, though in some cases it was used as a complement to flexibility when children were very young. In general, however, the possibility to vary start and finishing times of the job and take time off for errands was emphasized as a main solution. “I have a set schedule, but there is also some flexibility, and without that it would have been really tough,” says Annica, who works as a teacher. Like other mothers in the group, she and her partner share housework and care relatively equally, also when children are sick. Nevertheless, using CSC-leave is often a source of stress, mainly because it can cause problems for colleagues and pupils.

## 5 Conclusions

We found, in response to RQ 1, that employee- and employer-oriented flexibility tend to go hand-in-hand. The main contrasts are the boundaryless jobs – dominated by fathers – with high demands and high control both in time and strain dimensions, and the confined jobs – dominated by mothers – with high job demands but low job control and flexibility (both employee- and employer-oriented). For RQ2, we conclude that in both confined and boundaryless jobs the right to use CSC-leave is compromised: either parents worry that their leave causes problems or they bring work home to do while caring for the child. High flexibility also does not alleviate work-family conflict; instead, mothers with full-time work perceive more conflict in boundaryless jobs. Meanwhile, part-time work remains an important strategy for mothers to limit negative spillover. Parents in malleable jobs, with low demands and relatively high control in time and strain dimensions, report less problems in every aspect.

The typology emerging from the qualitative data analysis (RQ 3) further illustrates that flexibility is relational and the actual room to manoeuvre can be compromised both by employer demands and the partner’s job situation. Clearly, a lack of flexibility is problematic and in confined jobs female part-time work remains a main way of addressing work-family tensions. However, in boundaryless jobs, schedule control is offered in exchange for performance, commitment and availability and for this reason, fathers’ flexibility can require that their partners reduce work hours and take on more housework. At the same time, boundaryless jobs can allow for a household-level contract of equal sharing. For mothers, such agreements seem crucial, but in the Swedish context, demands for employer flexibility also clash with fathers’ ideals of involved parenthood.

## 6 Discussion

Flexible scheduling has emerged as a central theme both in policy debates and work-family research. The main thrust of our study was to go beyond the variable-oriented view to take a more contextual, holistic approach. Using a mixed-methods study of Swedish parents, we explored how work arrangements were constructed in terms of employee- and employer-oriented flexibility and how they related to work-family tensions, as reflected in quantitative correlations and parents’ own narratives.

The analysis suggests that flexibility is not just a tool that can be applied across contexts with similar effects. Rather, flexible scheduling emerges as an organizational logic that will affect employee-employer relations as well as families. A main finding is that employer- and employee-oriented flexibility go hand in hand, but that work arrangements differ radically between groups. In our cluster analysis, the jobs classified as confined were as common as those labelled boundaryless. The boundaryless jobs combine high levels of employee schedule control with high requirements for organizational flexibility, while confined jobs have low levels of both. Both clusters have high psychological work demands, but differ crucially regarding employees’ level of task control. At first glance, these clusters resemble the ‘good’ and ‘bad’ jobs as conceptualised in research on work and stress [23] and in the idea of flexible scheduling as a ‘win-win’ concept for employers and employees.

Our findings tell a different story. Regarding work-family conflict, both clusters perform worse than the malleable jobs, where employee control is high and employer demands low, both in time and strain dimensions. Most notably, parents in boundaryless jobs do not report less problems than those in confined jobs. Instead, work arrangements provide different challenges to different groups. Taking formal leave to care for sick children is problematic both in boundaryless and confined jobs. Meanwhile, parents in boundaryless jobs are prone to bring work home while caring for a sick child. Presumably, this is a way of coping with the problems of taking a leave and, more generally, to balance high demands from work and family. Despite such strategies, however, parents in boundaryless jobs do not perceive less work-family conflict than parents in confined jobs.

Overall, the links between gender, flexibility and work-family reconciliation are complex. In line with previous studies, we find that mothers have less access to schedule flexibility and job control than fathers. Additionally, we find that even with the same type of work arrangement mothers experience more work-family conflict than fathers and are more prone to work while caring for a sick child. In confined and malleable jobs, mothers also find it more problematic to take formal leave to care for a sick child. Here, boundaryless jobs provide an exception – perhaps because mothers with flexible scheduling (and portable work tasks) can mitigate the problem by working while caring. At the same time, mothers in full-time work experience more work-family conflict in boundaryless jobs than in confined jobs. The qualitative analysis further illustrates the importance of how the implications of employees flexible scheduling concerns also extend to their partner. Interestingly, this could imply both gendered outcomes and a possibility for change. For some, fathers’ employer flexibility create an inflexibility that limits mothers’ options, while other parents use flexible schedules to negotiate a contract of equal sharing of unpaid work.

Generally, however, parents tend to talk about schedule flexibility as a helpful tool, while organizational demands for flexibility are rarely problematized. In these narratives, flexibility emerges as a new psychological contract in which employee-oriented flexibility is a bonus or a gift that is traded for high organizational commitment, including constant availability. Previously, psychological researchers have discussed how the flexibilization of modern working life increases the need for boundary management to prevent negative health implications [4, 44, 45]. Our study is in line with these observations but further shows how problems of ‘boundaryless’ work are connected to organizational demands in a gender-segregated labour market and how family policy rights are challenged by this new logic. Arguably, then, the flexibilization of working life may foster a paradoxical notion of ’family-friendliness’ that reshapes rather than replaces ideal worker norms.

Several limitations should be mentioned. Cross-sectional survey data do not allow for inferences about causal relationship; the study is a description of the situation of individuals with different work arrangements and our ambition is not to stipulate the drivers behind these situations. Moreover, due to the varying response rates, the results may not reflect the situation of marginalized groups. While Sweden was considered a good case for studying flexibility in a dual-earner context, a single-country study may limit the generalizability of the findings. At the same time, our study seems timely considering the renewed interest in flexibility, gender and work-family reconciliation. Since the outbreak of the COVID-19 pandemic, we have seen an explosion of research on these issues [46–48]. These studies suggest that flexible working may not be enough to challenge traditional gender roles and that flexibility must be considered in relation to broader employment and family policies. Ideally, then, future research should approach these issues with cross-country comparative designs that can explore the importance of norms and institutions.

In terms of practice and policy, the results from our study strongly suggest that employers, trade unions and occupational health care services should strive to create ‘boundaries’ to ensure that flexibilization does not undermine standards of decent work. At the same time, we should not lose sight of the confined jobs, where a lack of flexibility and control can make it difficult for women to work full-time.

## Supplementary Material

Supplementary Material

## Footnotes

^1^
In previous research, it has been recognized that work-family conflict can take two directions, work interference with family (WIF) and family interference with work (FIW). We focus on WIF which is the most prevalent, and the most commonly studied, form of conflict. Also, work arrangements are more relevant to this type of conflict [16, 18, 40].

^2^
While a few previous studies have included employer requirements for flexibility in their analyses of flexibility and work-family conflict, these studies apply a variable-oriented approach without considering the context of flexible arrangement in terms of employer demands and employee control in time and strain dimensions [10, 11, 42].

^3^
Generally, research on work-family conflict report incongruent findings for gender [16, 32, 43]. Arguably, however, a lack of gender differences may hide the fact that women and/or mothers often adjust their work involvement to reduce frictions between work and care demands [31]. For example, several studies find that gender differences in work-family conflict increase when work hours are controlled for.

^4^
In Sweden, parents have a statutory right to reduce work hours with up to 25% until the youngest child is eight years old with a corresponding reduction in wages. However, parental leave compensation can be used flexibility to further reduce work hours and compensate for wage loss. The CSC-leave allows parents to stay home for up to 120 days per year with 80% income compensation to care for a sick child.

^5^
It should be noted that schedule control is not a statutory right in Sweden. It can be provided in collective agreement, often at the workplace level, but also through unilateral employer policies. At the same time, workers’ rights are backed up by central collective agreements which cover 90 percent of Swedish workplaces [49]. Ultimately, laws on working time and work environment provide a framework for negotiations between social parties. These include e.g., limits to work hours and overtime ceilings, rights to minimum time for rest etc..

^6^
Due to an initial mistake at Statistics Sweden, the sample had to be drawn twice. Because the first sample erroneously included parents who also had a child younger than 3, the survey was paused at an early stage and a new sample was drawn. The response rate for this sample was 35% but since some respondents from the first sample matched the inclusion criteria these were added to the final sample to increase the statistical power of the analyses. Analysis of non-response shows little difference between mothers and fathers. Individuals with compulsory education were underrepresented while those with tertiary education were overrepresented among the respondents. However, the share of individuals with secondary education did not differ substantially from that of the population. Also, immigrants/children of immigrants and individuals with very low incomes were underrepresented. Thus, the analyses are likely to capture the situation for parents with secondary and post-secondary education (together representing 75% of the population) but may not fully reflect the work-family arrangements of groups that are more marginalized on the labor market.

^7^
Further analyses show that in confined and boundaryless jobs, the gender difference is larger before controlling for workplace gender composition whereas in malleable jobs no significant gender difference appears even in bivariate regressions. Interaction variables testing for gender*workplace gender composition effects were not significant for any cluster. Thus, while work arrangements differ between workplaces with different gender mix, they do not differ between the minority and the majority gender.
