# Supplementary material for: A more equal deal? Employer-employee flexibility, gender and parents’ work-family tensions in Sweden
Source: Work. 2022 Nov 11;73(3):843–56. doi: 10.3233/WOR-210668 (PMC9697062; doi:10.3233/WOR-210668)
Supplement: Supplementary Material [file wor-73-wor210668-s001.docx]

Supplementary Table 1. Model-fit statistics for four latent class models.

|  | **L²** | **BIC(L²)** | **df** | **L² reduction (%)** | **p-value** |
| --- | --- | --- | --- | --- | --- |
| 1-Cluster | 449.37 | 364.709 | 11 | 0.0 | 0.000 |
| 2-Cluster | 87.52 | 41.342 | 6 | 80.5 | 0.000 |
| 3-Cluster | 6.64 | -1.060 | 1 | 98.5 | 0.010 |
| 4-Cluster | 0.003 | 30.790 | -4 |  |  |

Supplementary Table 2. Work hour categories by cluster and gender (%).

|  | **Confined jobs women** | **Confined jobs men** | **Boundaryless jobs women** | **Boundaryless jobs men** | **Malleable jobs women** | **Malleable jobs men** |
| --- | --- | --- | --- | --- | --- | --- |
| Part-time work | 35.4 | 6.9 | 13.9 | 3.2 | 25.7 | 8.0 |
| Full-time work | 52.0 | 72.5 | 49.2 | 44.5 | 65.6 | 77.3 |
| Long work hours | 12.6 | 20.6 | 36.8 | 52.3 | 8.7 | 14.7 |
